# Neurological telerehabilitation in the COVID-19 era – current perspectives through a bibliometric analysis

**DOI:** 10.3389/fneur.2023.1227846

**Published:** 2023-09-19

**Authors:** Lúcia Aparecida Lebioda, Bruno Pedroso, Marlon Estevam Camilo dos Santos, Guilherme Moreira Caetano Pinto, Leonardo Christiaan Welling

**Affiliations:** Graduate Program in Health Sciences, Universidade Estadual de Ponta Grossa, Ponta Grossa, Brazil

**Keywords:** SARS-CoV, telerehabilitation, COVID-19, stroke, rehabilitation

## Abstract

**Objective:**

To identify bibliometric parameters and research trends regarding to telerehabilitation of patients with stroke in the COVID-19 era.

**Methodology:**

This is an integrative review carried out in the Scopus database, from June to July 2021, through the Biblioshiny graphical interface, provided by the Bibliometrix program. The search terms used were “Stroke,” “COVID-19” and “Telerehabilitation.” Results were filtered by publication date from 2019 onwards. No language restrictions were imposed.

**Results:**

Twenty two articles were included in the study and the results were presented in the form of figures demonstrating that the journal Frontiers in Neurology was the one with the most relevant studies and the author with the highest number of citations. The Brazilian Academy of Neurology was the institution with the greatest number of studies and China ranked first as the country with the greatest scientific production. The authors used recent references in their work. A thematic map showed the centrality and density of the words presented and, finally, a three-field graph showed a strong intimacy between countries, keywords and authors.

**Conclusion:**

A greater interest in the subject was observed in China with greater relevance of journals and institutions focused on neurology. However, despite telerehabilitation being an effective alternative in the context of the pandemic, few studies have explored this modality.

## Introduction

The outbreak caused by the SARS-COV-2 virus and its high transmission rates resulted in a situation that was declared a pandemic by the World Health Organization (WHO) on March 11^th^, 2020 (1). In order to reduce the transmission of the pathogen and contribute to social distancing, several health services, such as rehabilitation centers, became unavailable or had their activities suspended for an indefinite period (2).

Rehabilitation centers provide care for various pathologies, including cerebrovascular accident (CVA), which is considered the first cause of disability and the fifth cause of death in the United States (3, 4). With a high incidence rate, the Ministry of Health (MS) predicts that one in four people may suffer an ischemic or hemorrhagic event throughout their lives5. Stroke is linked to risk factors that include hypertension, diabetes mellitus, hypercholesterolemia, physical inactivity, obesity, smoking and genetic factors (3–5).

In addition to requiring immediate follow-up, patients with neurological or immunosuppressed diseases have greater complications when infected with COVID-19 (6). Early rehabilitation is recommended in order to promote recovery, increase functional independence and improve quality of life after a stroke (7, 8). In addition, ensuring immediate access to rehabilitation for these patients is associated to neuroplasticity benefits, which refer to major reorganizational changes in the brain with consequent gains (9).

However, poor adherence to rehabilitation programs is a common problem among individuals who have suffered a stroke. This poor adherence is related to numerous factors, including environmental factors such as cost, accessibility and transportation. Increasing the effectiveness of treatment adherence interventions can have a positive and significant impact on the recovery of these individuals. In addition, there is evidence to suggest that adherence can be improved through motivational interventions, behavioral change strategies and through the use of multimedia, demonstrating that technology can work in favor of rehabilitation (8).

In addition to requiring immediate follow-up, patients with neurological or immunosuppressed diseases have greater complications when infected with COVID-19, with that and with the restrictions imposed on rehabilitation centers, the outbreak of the disease required a rapid digital revolution (6). One of the most viable forms of treatment at this time is telerehabilitation, which consists of providing rehabilitation services through information and communication technologies (10).

Telerehabilitation can occur through a variety of technological options such as telephone, videoconferencing, virtual reality programs, applications and software (11). Consultations may include assessment, diagnosis, goal setting, therapy, education and monitoring (10). The practice of rehabilitation by technological means has been discussed for at least three decades, due to the lack of accessibility for some patients, difficulty in moving, distance and severity of cases (11).

Telestroke programs, an audio-visual communication network that provide the foundation for a model of collaborative interprofessional care focused on acute stroke patients, can increase patients’ access to treatment and generate ongoing benefits beyond having been demonstrated as a medical practice. Safe and effective, providing a positive clinical impact on public health (4, 12).

However, there are still some barriers regarding to the most appropriate models or tools for the rehabilitation of these patients who suffer from stroke sequelae (11). Evaluating the interest and contribution of the scientific community, in terms of publications, can help in the knowledge about the subject, institutions that propose this rehabilitation and journals that show interest in publishing and cooperating with the collection of studies that focus on the telerehabilitation of stroke patients. In addition, considering the increase in the number of people who would benefit from telerehabilitation, especially after the suspension of rehabilitation centers and the social distancing imposed by the pandemic and the growing impact of technology on rehabilitation services, the objective of this review is to identify the Bibliometric parameters and research trends regarding to telerehabilitation of stroke patients in the COVID-19 era.

## Materials and methods

The problem that determined the development of the present research was the following: “How are the research trends facing telerehabilitation in patients with stroke in the COVID-19 era?”

This is an integrative literature review, carried out through a bibliometric study, whose purpose is to gather and synthesize research results in an orderly manner, contributing to a better understanding of relevant topics.

The definitive bibliographic research of the study took place from June to July 2021. The data collection was carried out through a search in the Scopus database. As descriptors, “Stroke,” “COVID-19” and “Telerehabilitation” were used, connected through the Boolean operator “AND.” The results were filtered by publication date, starting from the year 2019. No language restriction was imposed, and the search terms were used in English due to the fact that indexing in the Scopus database invariably requires the title in English and abstract.

The database search was performed by a single researcher. After the search, the documents were submitted to the analysis of the information provided by the database. Subsequently, findings whose title, keywords and/or abstract did not address the researched topic were eliminated. After thorough evaluation, the documents were exported from the database in BIBtex format and transferred to the Bibliometrix program. For the comprehensive analysis of scientific mapping, the Biblioshiny graphical interface was used, provided by the Bibliometrix program, in order to import and manage data (13).

The Biblioshiny program allows users to perform pertinent bibliometric and visual analyzes based on an interactive web interface (12). The base metadata included resources such as general data, production by year, publications by journal, most cited journals, most cited articles, institutions with the highest production, country of corresponding author, production by country, spectroscopy of references, cloud of words, growth of terms, thematic map, dendrogram, co-occurrence network, co-citation network and three-field plot (country/term/author).

## Results

**Figure 1:**
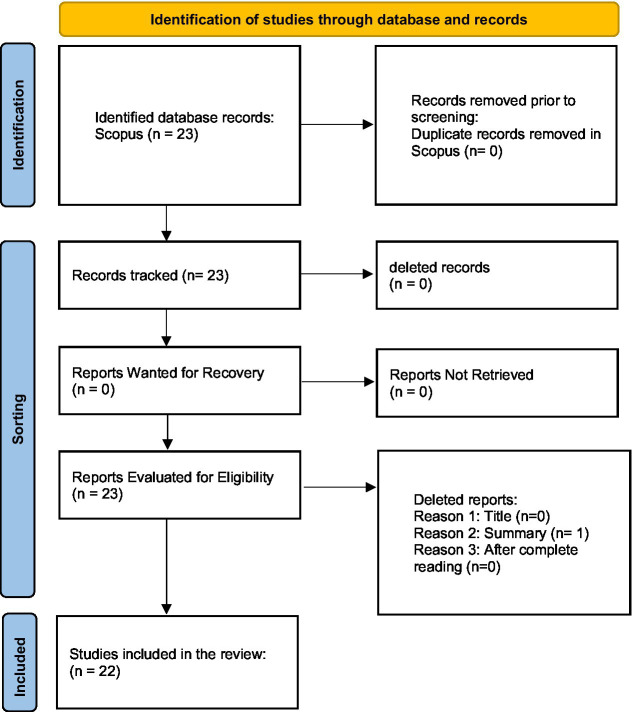
Flowchart for selecting studies, PRISMA – SsC (2020; Doi: https://doi.org/10.1136/bmj.n71).

The electronic search strategy resulted in 23 articles. After a careful and interpretative analysis of the studies on the subject, one study was excluded for being outside the scope of the present research and 22 articles contemplated the research objective and were included in the present Flowchart study. Figure 1 presents the flowchart for the selection of studies.

**Figure 2:**
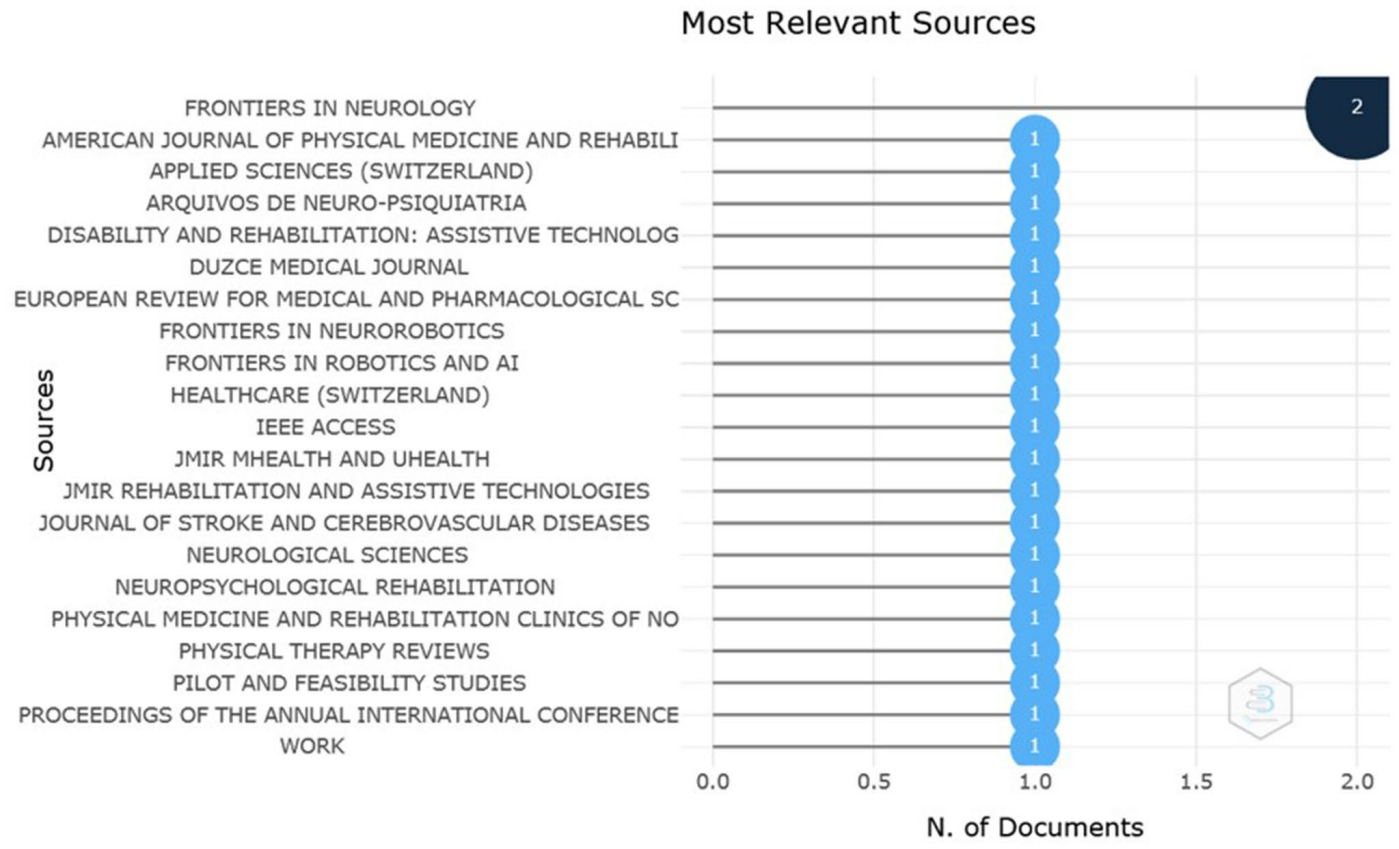
Journals and their respective scientific productions; Source: Scopus/Biblioshiny.

Of the included studies, eight were published in 2020 and 14 in 2021. Publications referring to telerehabilitation methods in times of COVID-19 were scattered. Figure 2 shows the most relevant sources and the number of documents published by each one of them. The journal Frontiers in Neurology was the journal with the highest number of publications. The other journals had only one study published on the topic researched.

**Figure 3:**
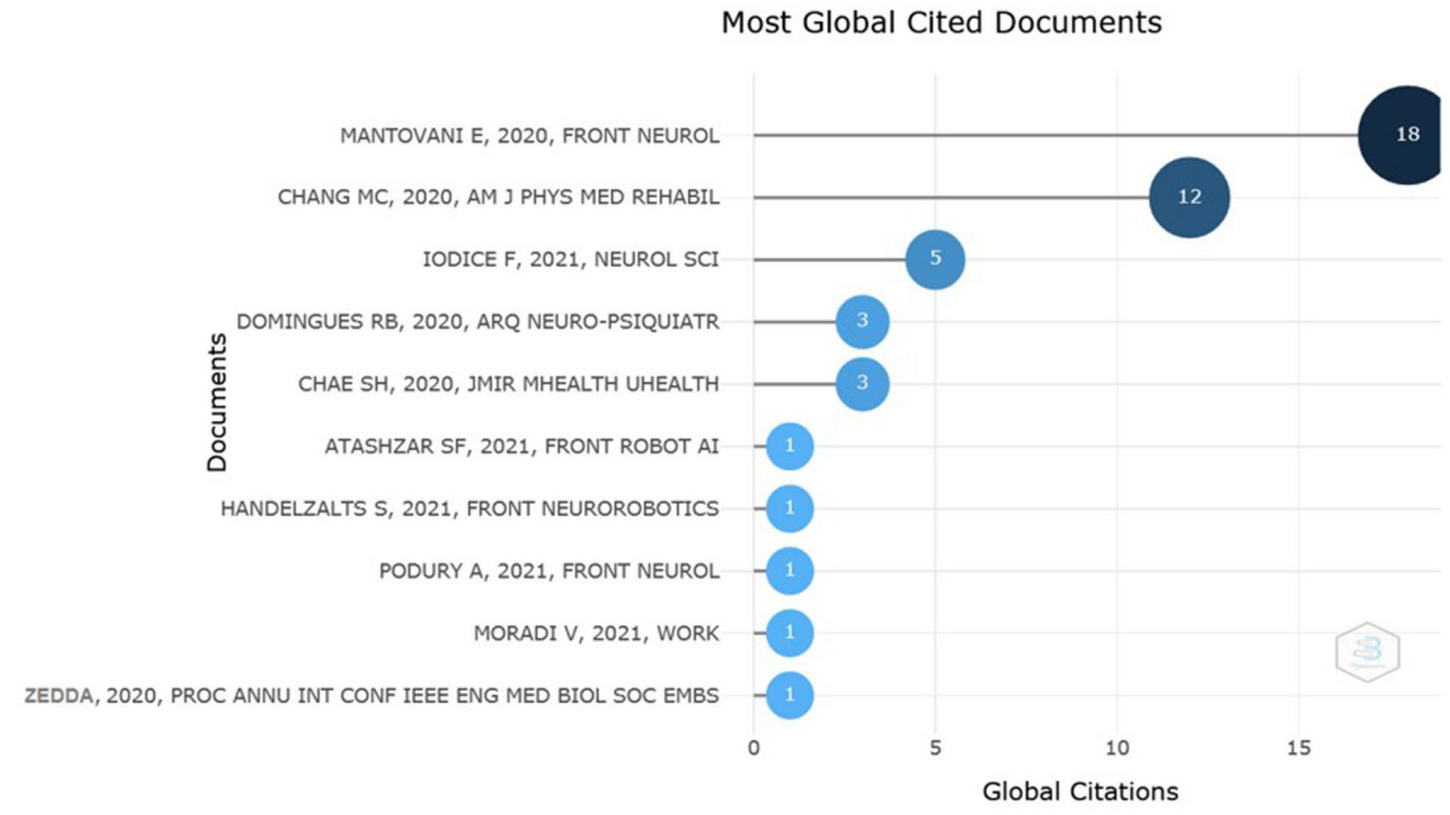
Most cited documents globally. Authors, year, journal and number of citations. Source: Scopus/Biblioshin.

Another relevant data refers to the most cited documents globally. Figure 3 indicates the first or only author of each work, followed by the year of publication, the journal in which it was published and the number of citations corresponding to each author. In this figure, it can be seen that the most cited author had his article published in Frontiers in Neurology and received 18 citations until the moment this research was carried out.

**Figure 4:**
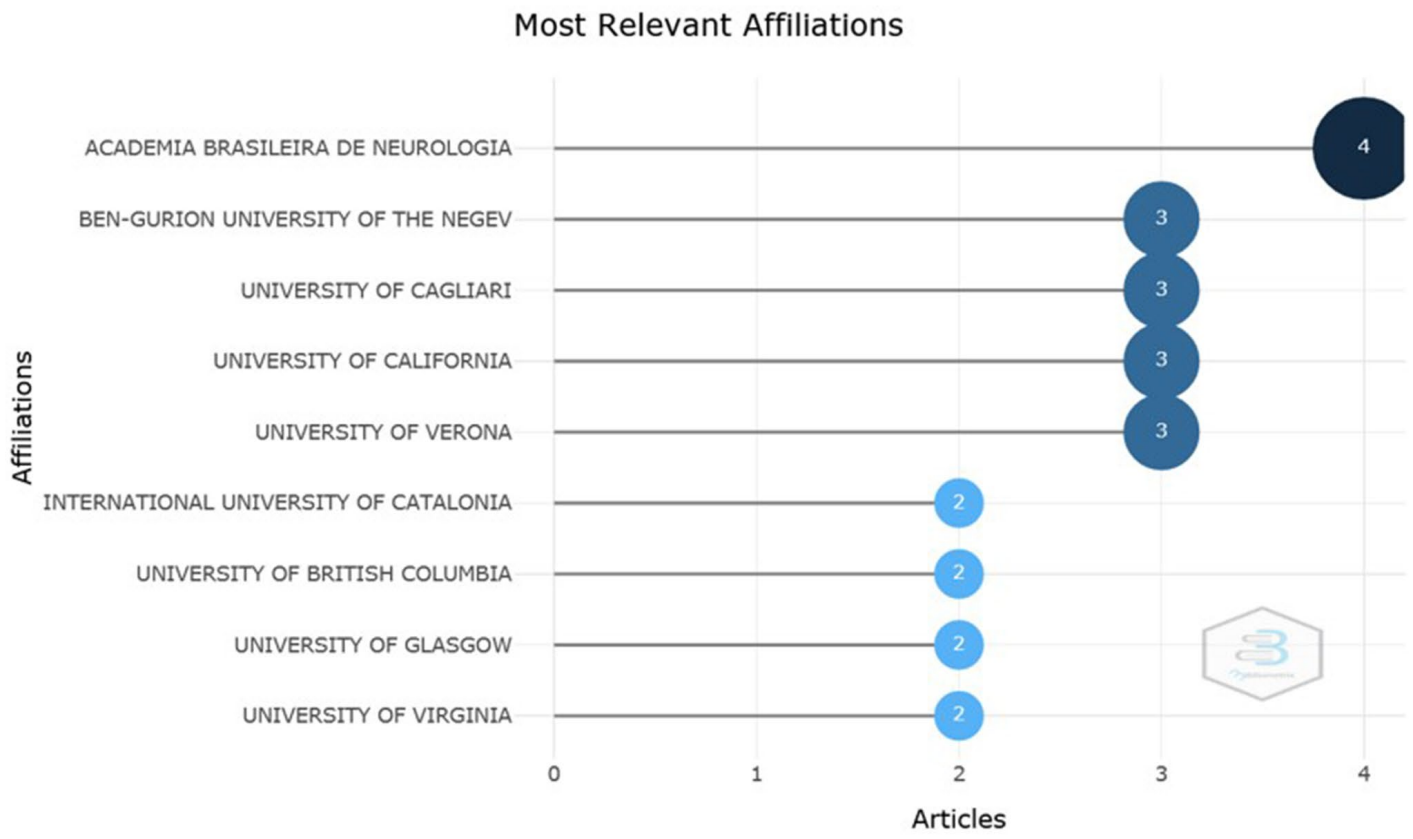
Institutions with the highest production. Most relevant affiliations according to the number of productions. Source: Scopus/Biblioshiny.

According to the collection of metadata found, Figure 4 highlights the institutions with the highest production and the number of articles produced. It is observed that the Brazilian Academy of Neurology was the institution with the highest production of articles, producing a total of four papers. In sequence, one can observe the Bem-Gurion University of the Negev, University of Cagliary, University of California and the University of Verona with three published works. The other listed universities obtained a total of two articles produced and published.

**Figure 5:**
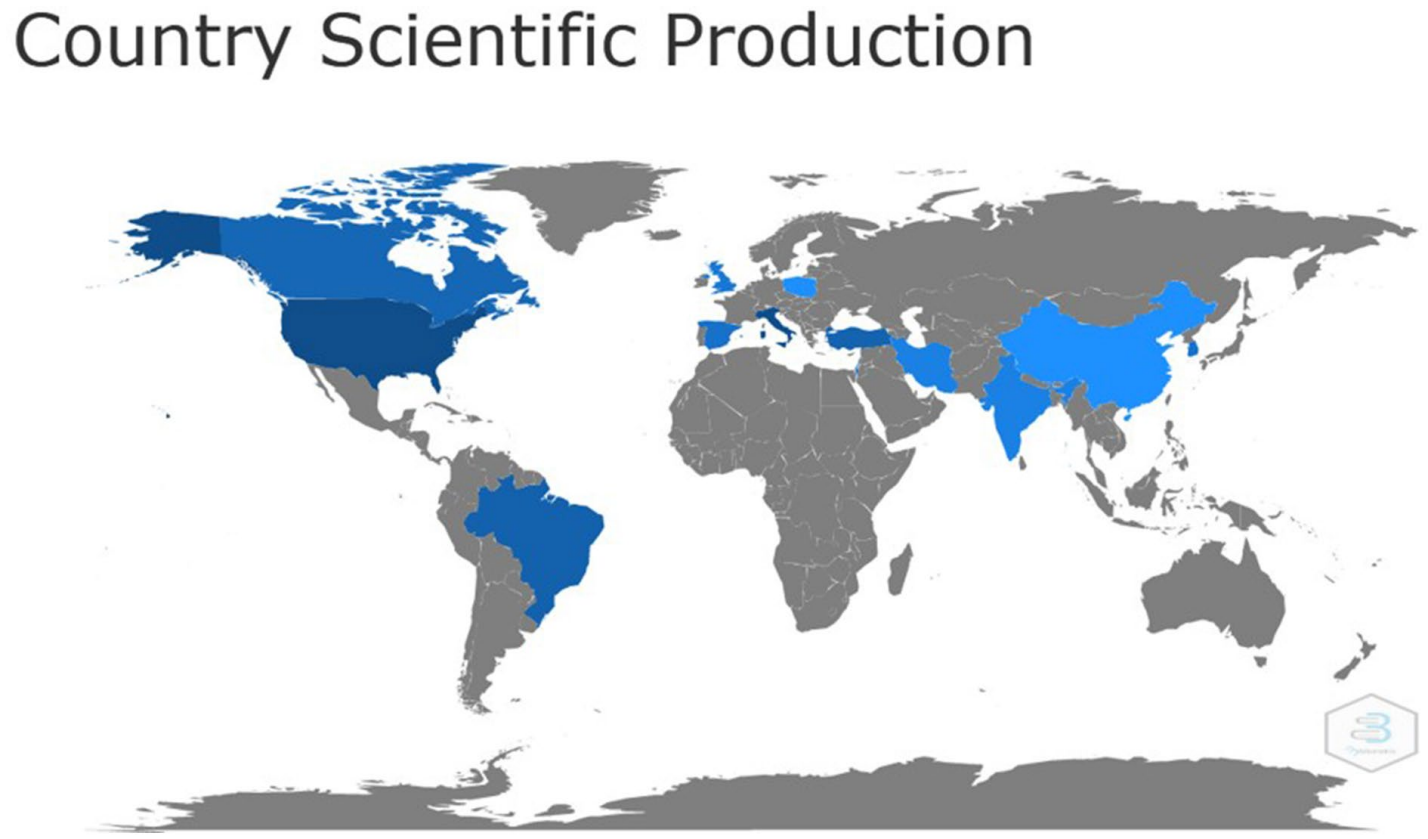
World map demonstrating the scientific contribution of countries. The darker blue color indicate a higher incidence of scientific production in the country. There is evidence of a greater contribution from North America, South America and the Asian continent. Source: Scopus / Biblioshiny.

Mapping the scientific collaboration of countries with the highest production, Figure 5 shows the world map according to publications between 2019 and 2021. This map provides, in blue color, the countries that showed the greatest interest in the researched topic. A greater interest can be observed in countries of the American continent, dispersed mainly in North America, and in the Asian continent.

**Figure 6:**
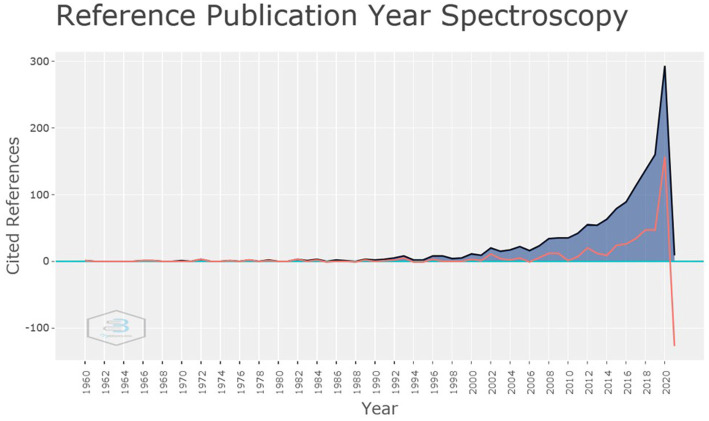
Spectroscopy of reference years. Years of reference used by authors. Upline after 2019. Source: Scopus/Biblioshiny.

A graph, called reference spectroscopy, shows the years of literature used by the authors. In this graph, a sharp increase can be seen from the year 2019 (Figure 6).

Another way of analyzing the studies is through a thematic map, presented in the form of quadrants. The X axis represents density, that is, it measures the internal strength of a cluster network and is a measure of theme development. The Y axis represents the centrality, that is, the degree of interaction of a network cluster compared to others.

**Figure 7:**
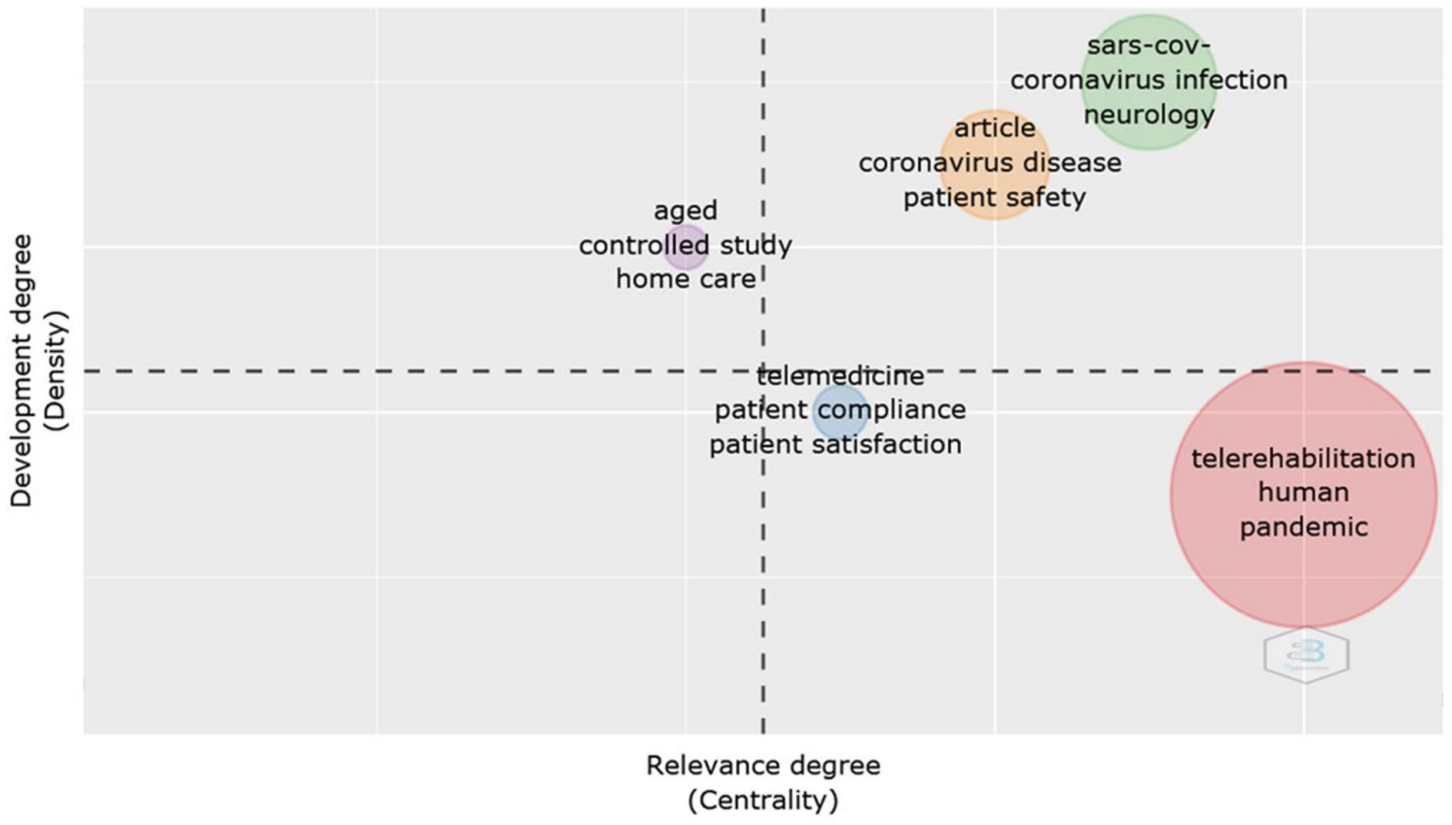
Thematic Map. Presentation of interdisciplinary themes (1 cluster), engine themes (2 clusters), theoretical themes (2 clusters), emerging or declining themes (no cluster). Source: Scopus/Biblioshiny.

In Figure 7, five clusters can be identified. The first quadrant, upper left, presents a cluster of high centrality and high density that included the words “aged,” “controlled study” and “home care.” These are niche topics, interdisciplinary topics that do not specifically fit into any of the other quadrants. In the upper right quadrant, the motor themes are presented, which drive the production of the studied area, in this, two clusters are observed, one of them includes the words “SARS-CoV, coronavirus infection and neurology” and another “article, coronavirus disease and patient safety.” In the lower right are the basic themes and in this quadrant two clusters are also presented, one with high density and low centrality, with the words “telerehabilitation, human and pandemic” and another with high centrality and density, with the words telemedicine, patient compliance and patient satisfaction.” These are more theoretical and underpin the research. Finishing the quadrants, the last one, in the lower left corner, does not present any cluster. In this, emerging themes and themes that are in decline would be grouped together. It is natural that themes like these do not appear in a survey that covers a short period of time, as is the case of this one.

**Figure 8:**
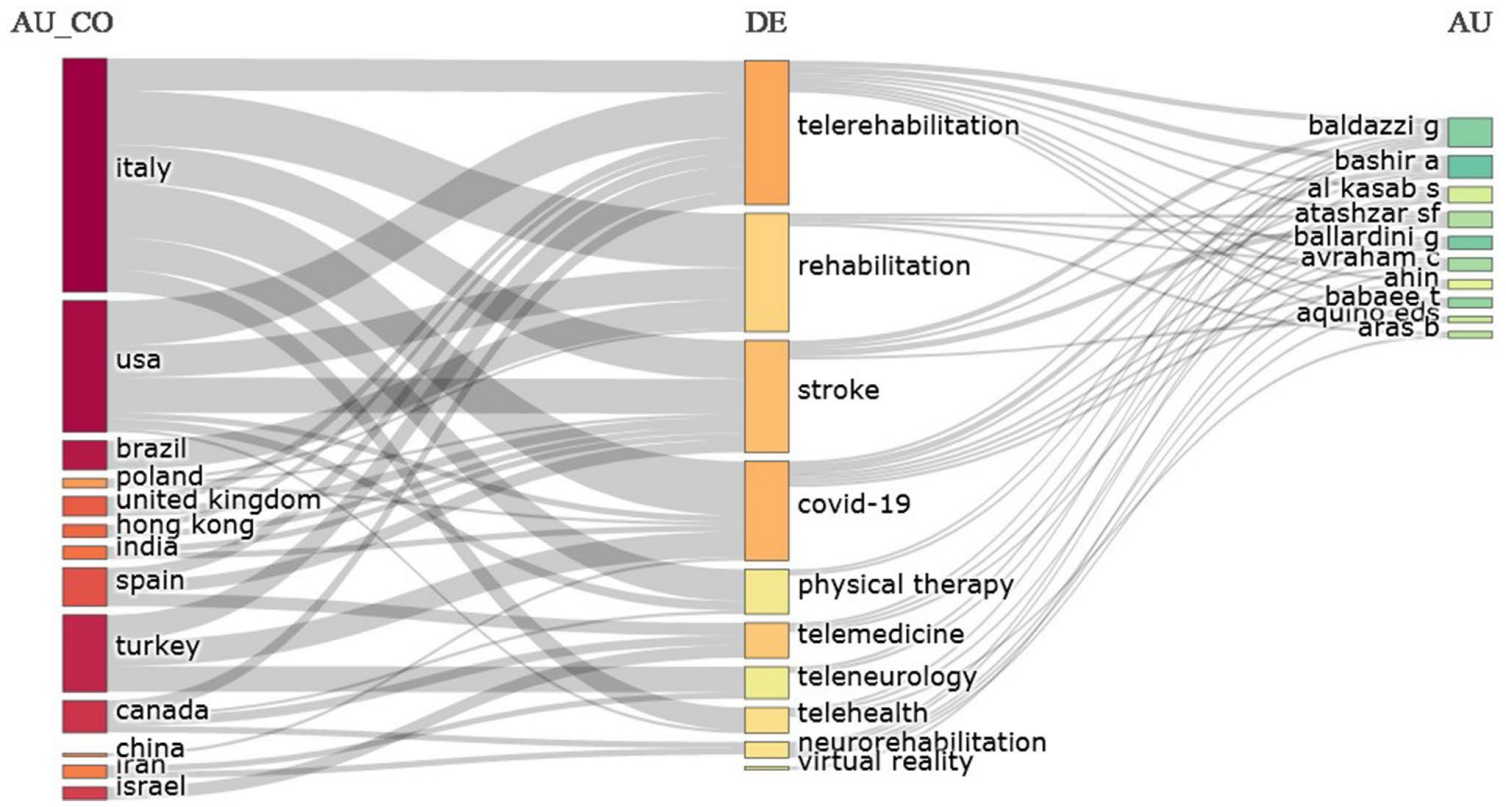
Three-field graph representing the relationship between countries, keywords and top authors. The higher the rectangle, the greater the element’s relation to the other fields studied. Source: Scopus/Biblioshiny.

Finally, a three-field graph represents the relationships between countries, keywords and main authors. The rectangle diagram illustrates the main elements. The larger the rectangle (higher) the greater the relation that the shown element has (Figure 8).

## Discussion

Bibliometric analysis has lately become popular, applying metrology characteristics of the literature to measure the contribution of certain areas of research, predicting detailed trends and strongly collaborating with the prevention and treatment of diseases (14). However, there is still a shortage of studies using this methodology focusing on telerehabilitation of stroke patients in the COVID-19 era.

In times of technological advances, the COVID-19 outbreak demanded a reinvention from the world (15). It is known that research through methodologies and scientific approaches are crucial in the role of coping with and controlling the disease. To this end, recent studies, in different institutions, have been initiated and implemented in a comprehensive way since the appearance of the virus in 2019 (16).

The findings reveal that COVID-19 can affect the circulatory system, which contributes to the sum of pathological events that can affect the central nervous system (CNS) and lead to neurological problems (17). The disease is also associated to deficits in coagulation functions, formation of thrombi and microthrombi in extremities (lower limbs) and in central regions such as the brain (18).

Furthermore, it is documented that SARS-CoV-2 has neuroinvasive properties that can cause diseases such as stroke, Guillain-Barré syndrome and critically ill polyneuropathy, confirming a neurological involvement (19). These findings may justify the interest of journals and institutions related to neurology, such as Frontiers in Neurology, which appeared first as a most relevant source, with two articles published (Figure 2) and was also the journal in which the work with the highest number of citations was published (Figure 3). Another interest related to neurology is shown in Figure 4, in which the Brazilian Academy of Neurology appeared at the top of the ranking of institutions with the greatest production, with four articles produced.

However, the number of publications related to the topic, the citations of documents already published and the production of research institutions is still low. The initial hypothesis would be that the set of information about treatment aimed at telerehabilitation had increased, taking into account the emergence of the topic. This fact, which differs from publications related only to COVID-19, as a way to fight the disease and not its consequences, in which the contents are published daily (20).

Regarding to countries with greater scientific production, in Figure 5 the darker blue color indicates a higher collaboration rate. In this sense, it can be observed that the American continent was the largest producer, especially North America, with the United States of America being the most prominent country. The United States of America is a strong research power and the country was considered, in other studies, as the main collaborating country occupying the leadership of research related to COVID-19 (14–21). The fact may happen as a result of superior conditions for medical research, including funding, advanced equipment and professional researchers (14).

In studies carried out by Chahrour et al. and Dehghanbanadaki et al. the China was among the most productive countries (21, 22). This may be related to the fact that China was the birthplace of the pandemic and it is a country that is home to more than 3.61 million licensed physicians and, therefore, it is not surprising that most of the most relevant publications are attributed to the chinese institutions (22).

These two countries, China and the United States of America, occupy the first places in the ranking of countries with the highest number of people affected by COVID-19. Table 1 shows the COVID-19 situation according to the countries with the highest number of cases.

**Table 1:** Situation of countries according to the number of people affected by COVID-19, according to WHO.

Country	Cases – cumulative total	Deaths – accumulated total
The USA	103,436,829	1,127,152
China	99,296,816	121,536
India	44,994,955	531,915
France	38,997,490	167,985
Germany	38,437,756	174,979
Brazil	37,693,506	704,320
Japan	33,803,572	74,694
Republic of Korea	32,611,509	35,159
Italy	25,904,951	190,942
UK	24,641,596	228,144

Adapted from: https://covid19.who.int/table.

It is interesting to note that both countries that had the highest rates of involvement by COVID-19 were also the countries with the greatest scientific production regarding to telerehabilitation. This suggests a growing interest from countries in post-stroke telerehabilitation during the COVID-19 pandemic.

Another relevant aspect that may have helped propel these two countries forward is their technological competitiveness and emerging technologies. At the end of World War II, the United States dominated all spheres: commercial, monetary-financial, military and technological. However, in the last 30 years China has become a technological superpower, justifying its equality with the United States (23). In addition, they are countries that are among the leaders in publications associated to technological research (24, 25). Undoubtedly, the data obtained in the present study demonstrate that these two countries are central and influential in research, helping to establish a solid base for the development of future treatments.

Regarding to the contemporaneity of the references used in the studies presented in this review, through the spectroscopy graph, Figure 6 demonstrates that the vast majority of articles used references from recent years, with a noticeable ascending line from the year 2014. It is known that the use of telemedicine is expanding and a possible increase of 20 to 50% in the rates of use of these resources is foreseen, including its subsets as is the case of Telestroke (26). Another fact that may justify the finding of recent references is that COVID-19 emerged a short time ago and is a topic that is still on the rise (27).

With regard to the thematic map, shown in Figure 7, it has already been mentioned that COVID-19 affects several organs and systems. Due to this fact, the disease requires an interdisciplinary approach, covering all branches of medicine, including the virtual approach, through telemedicine (28). The driving themes refer to the coronavirus disease and the basic themes to telerehabilitation. These are topics that deserve to be studied and aim at understanding the pathology, improving the quality of life and psychological well-being, mainly aimed at the rehabilitation of chronic conditions (11). It emphasizes the need for more studies that can leverage the discussion on the importance of research aimed at telerehabilitation.

Another relevant data is the analysis of countries, main authors and keywords. Figure 8, indicates five countries (Italy, United States, Turkey, Brazil and Canada), four keywords (telerehabilitation, rehabilitation, stroke and COVID-19) and two authors (Baldazzi G and Bashir) who were most closely linked to the key research topics related to telerehabilitation of stroke patients in the COVID-19 era. In this finding, it is observed that Italy appears strongly related to the other two topics, which indicates a multiparty collaboration, in which several countries are contributing to research related to the subject studied (14).

In the bibliometric study carried out by He et al. (29) which aimed at evaluating emerging trends in virtual reality in cognitive rehabilitation, the authors obtained contributions from countries such as the United States, Italy, United Kingdom, China, Germany, Australia, Spain, Canada, France and the Netherlands. They are different countries in terms of population, financial resources and geographic distribution. However, they are countries that are similar in their strong interest in research, mainly the United States and Italy that emerged in both studies, occupying the first two positions. It is estimated that, in 2050, the number of individuals with a disability in the United States will reach 64 million, justifying the interest in the subject (29). On the other hand, it was expected that China, due to the higher population number and the large volume of studies published after COVID-19, would obtain one of the first places in this analysis. This finding demonstrates that the country has not been very interested in the researched topic (29).

Telerehabilitation is an opportunity to continue the treatment of patients who have been discharged from the hospital. We highlight the effectiveness of using telerehabilitation in the physiotherapy service which, through technology, can individualize care, promote health education, carry out assessments, outline treatment plans and monitor the progress of rehabilitation through feedback and continuous supervision. Furthermore, it is noteworthy that patients can benefit from remote care, as telerehabilitation can help reduce hospitalization and readmission rates, offer immediate access to outpatient services and promote better health outcomes and, consequently, better quality of life (30). COVID-19 brought us countless harms, but it left lessons learned. That the use of telerehabilitation be encouraged in order to benefit the community that needs care.

As far as we know, this is the first bibliometric analysis of research on telerehabilitation of stroke patients in the COVID-19 era, in which it was possible to demonstrate a real and current situation on the subject. However, the present study has some limitations, such as the use of a single database (SCOPUS). The choice of the Scopus database is justified because it comprises the largest number of journals indexed among the main databases of excellence and worldwide recognition. Furthermore, it was decided not to use additional databases because the non-unification of databases in the use of the Bibliometrix results in severe restrictions. Also, the research covered a short period of time, which consequently resulted in the scarcity of studies related to the topic under examination. However, this was due to the recent appearance of COVID-19, and the research covered the entire period since the appearance of the aforementioned virus. In addition, mention should be made of the lack of registration of a pre-planned protocol for carrying out the study.

Regardless of the limitations mentioned above, the objective of the study was achieved, and it is expected that new research can be based on the present study to carry out future investigations regarding the theme addressed.

## Conclusion

The results of this bibliometric review document demonstrate that most of the research related to telerehabilitation of stroke patients in the COVID-19 era was carried out and reported in China, with greater interest from journals and institutions focused on neurology and, in this sense, there was no a predominant journal that concentrated the publications, but the review revealed an institution with a greater number of studies that demonstrate greater interest in the subject and a work that stood out for the number of citations received. Other countries such as Canada, Brazil, the United States of America and Italy were precursors in this research. The thematic map highlighted the importance of an interdisciplinary approach, due to the various systems affected by COVID-19. However, despite telerehabilitation being an effective alternative in the context of the pandemic, few studies have explored this modality.

## Data availability statement

The original contributions presented in the study are included in the article/supplementary material, further inquiries can be directed to the corresponding author.

## Author contributions

LL: definição do tema, desenvolvimento da pesquisa por meio da revisão integrativa e escrita do artigo. BP: orientação, correção e análise dos dados através da interface gráfica Biblioshiny, fornecida pelo programa Bibliometrix. MS: auxílio na escrita e formatação do artigo. LW: orientação na definição do tema de pesquisa, correção e tradução do artigo. All authors contributed to the article and approved the submitted version.

## Conflict of interest

The authors declare that the research was conducted in the absence of any commercial or financial relationships that could be construed as a potential conflict of interest.

## Publisher’s note

All claims expressed in this article are solely those of the authors and do not necessarily represent those of their affiliated organizations, or those of the publisher, the editors and the reviewers. Any product that may be evaluated in this article, or claim that may be made by its manufacturer, is not guaranteed or endorsed by the publisher.
